# Tuning Cancer Fate: Tumor Microenvironment's Role in Cancer Stem Cell Quiescence and Reawakening

**DOI:** 10.3389/fimmu.2020.02166

**Published:** 2020-10-21

**Authors:** Antonella Sistigu, Martina Musella, Claudia Galassi, Ilio Vitale, Ruggero De Maria

**Affiliations:** ^1^Istituto di Patologia Generale, Università Cattolica del Sacro Cuore, Rome, Italy; ^2^Tumor Immunology and Immunotherapy Unit, IRCCS Regina Elena National Cancer Institute, Rome, Italy; ^3^IIGM - Italian Institute for Genomic Medicine, c/o IRCSS Candiolo (TO), Candiolo, Italy; ^4^Candiolo Cancer Institute, FPO - IRCCS, Candiolo, Italy; ^5^Fondazione Policlinico Universitario “A. Gemelli” - IRCCS, Rome, Italy

**Keywords:** tumor microenvironment, cancer stem cells (CSC), disseminated cancer cells (DCC), reawakening, dormancy, immunoediting of cancer, immune escape, tumor evolution

## Abstract

Cancer cell dormancy is a common feature of human tumors and represents a major clinical barrier to the long-term efficacy of anticancer therapies. Dormant cancer cells, either in primary tumors or disseminated in secondary organs, may reawaken and relapse into a more aggressive disease. The mechanisms underpinning dormancy entry and exit strongly resemble those governing cancer cell stemness and include intrinsic and contextual cues. Cellular and molecular components of the tumor microenvironment persistently interact with cancer cells. This dialog is highly dynamic, as it evolves over time and space, strongly cooperates with intrinsic cell nets, and governs cancer cell features (like quiescence and stemness) and fate (survival and outgrowth). Therefore, there is a need for deeper insight into the biology of dormant cancer (stem) cells and the mechanisms regulating the equilibrium quiescence-*versus*-proliferation are vital in our pursuit of new therapeutic opportunities to prevent cancer from recurring. Here, we review and discuss microenvironmental regulations of cancer dormancy and its parallels with cancer stemness, and offer insights into the therapeutic strategies adopted to prevent a lethal recurrence, by either eradicating resident dormant cancer (stem) cells or maintaining them in a dormant state.

## Introduction

Despite the many noteworthy improvements in early diagnosis and treatment of primary tumors in recent years, in many cases, cancer patients develop distant metastases that, almost invariably, portend a poor prognosis. The current view is that metastatic relapse is caused by the reawakening of disseminated cancer cells (DCCs) from a dormant and asymptomatic state, after a time-lag lasting from a few months to several years.

**Figure 1:**
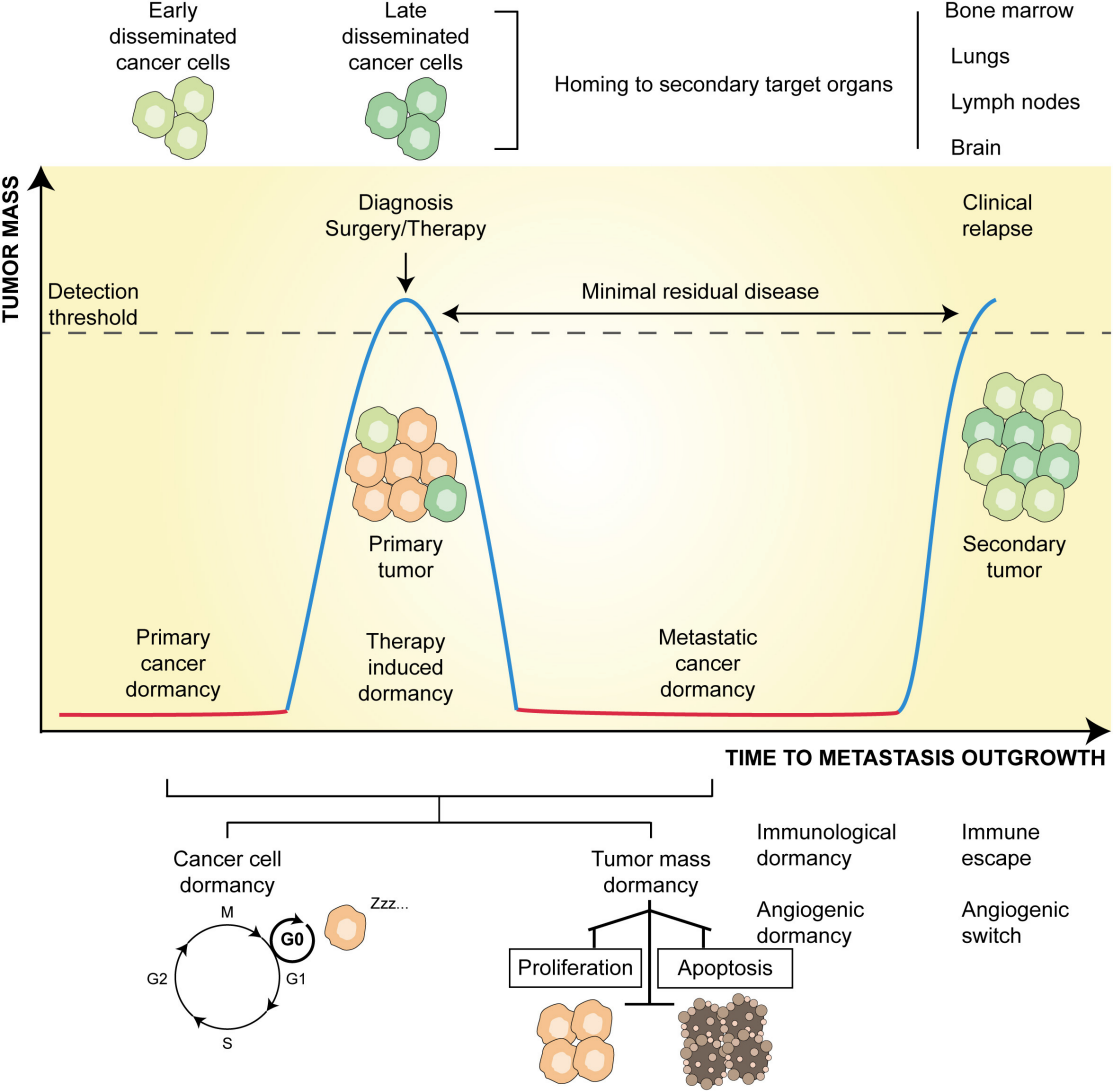
Principles and temporal course of cancer dormancy. Along with primary tumor development, a state of dormancy (red line) allows the survival of microscopic bulk cancer cells. The progressive evolution of the tumor, accumulating genetic and epigenetic changes, and its microenvironment, molding immune, and angiogenic contextures, eventually lead to tumor outgrowth (blue line). At this time, early disseminated cancer cells may develop and home to metastatic sites (mainly bone marrow, lungs, lymph nodes, and brain). Following treatment (either surgery or therapy or both) leading to tumor regression, resistant cells may persist latent (red line) and constitute an undetectable minimal residual disease. At this time, late disseminated cancer cells may develop and join early counterparts at secondary organs. After a time lag, which can last from a few months to many years, these disseminated cells may overgrow and give rise to metastatic clinical relapse (blue line). Dormancy might be due to solitary cells entered into a G_0_ phase of cell cycle arrest (cancer cell dormancy) or to the equilibrium between the rate of proliferation and apoptosis (tumor mass dormancy) mainly influenced by angiogenic and immunological cues.

Cancer dormancy is broadly defined as a stalled phase of cancer progression during which single cancer cells or microscopic tumor bulks remain clinically undetectable, yet retain the ability to progress into overt disease (1). Pristine mentions of cancer dormancy date back to the 1950s, when clinicians hypothesized that dormancy could explain cases of relapse observed several years after post-surgical and post-therapy remission (2). Nowadays, it is well-proven that dormancy is an adaptive and protective mechanism that malignant cells adopt to survive stress conditions of the tumor microenvironment (TME) (3). Moreover, cancer dormancy is considered a crucial part of the natural history of cancer evolution, irrespective of whether it occurs during primary tumor development (primary dormancy) or metastatic colonization (metastatic dormancy) (4) (Figure 1). In this setting, if the TME is growth permissive, cancer cells proliferate and give rise to overt diseases. If instead, the TME is not-permissive, cancer cells either are eradicated via the activation of regulated cell death or an irreversible proliferative arrest known as cellular senescence or survive by entering reversible dormancy. Dormant cells could then contribute to disease evolution by increasing their fitness via enforcement of genetic and epigenetic editing (5), and/or by promoting the remodeling of the TME, which then becomes “fertile soil” for outgrowth (3).

Three additional layers of complexity are emerging in the field of cancer dormancy, all of which have therapeutic relevance. First, at the molecular level, both the entry to and exit from dormancy are finely regulated by the cooperative action of cellular and molecular components of the TME (3). Of note, these contextual cues trigger a multitude of dormancy inducing signaling, and almost all overlap with those that induce cancer stemness (6, 7). This is supported by the evidence that cancer stem cells (CSCs)—the subset of cancer cells endowed with self-renewal ability, therapy-resistance, and immune evasion (8–10)—may switch between dormant and proliferative states (6, 7), resulting in an increased metastatic potential (11). Second, at the mechanistic level, tumor dormancy encompasses cellular dormancy (i.e., the condition in which solitary cancer cells temporarily arrest their cell cycle), and tumor mass dormancy, which refers to the condition in which clusters of indolent malignant cells enter a state of balanced proliferation/apoptosis rate (1). This balance, which prevents a tumor from increasing in size, seems to rely on (i) the absence of new vessel sprouting (so-called angiogenic dormancy) (3, 12), and (ii) immunosurveillance (so-called immunologic dormancy) (3, 13, 14). Finally, even though cellular senescence is widely considered as an irreversible and persistent cell cycle arrest (15), instances of reversible senescence and a causal link of the latter to disease recurrence have also been reported (7, 16).

In this study, we first describe the process of metastasization and the experimental models developed to study cancer dormancy and then discuss the role of the TME factors in regulating cancer dormancy and reawakening at metastatic sites. In particular, we focus on the intimate cooperation between different TME signals as we cover the complex relationship between immune-mediated dormancy and dormancy-mediated immune escape. During these discussions, we highlight the striking parallels between cancer dormancy and cancer stemness and summarize the current use of and ensuing therapeutic opportunities to prevent the occurrence of life-threatening metastatic relapse.

## Metastasis: Models, Evolution and Dormancy

It is estimated that metastatic relapse is responsible for as much as 90% of cancer-related deaths (17, 18). This is ascribed to the fact that progressing metastases rapidly become incurable, spread to additional sites, and compromise the function of vital organs (17). The clinical importance of cancer metastasis has been undeniable since the recognition of cancer as a disease, which has fostered massive experimental efforts to understand its origins and nature (19).

Taking stock of the increasingly large body of research to date, metastasis can be depicted as a sequential, multi-step process collectively conceptualized as the invasion-metastasis cascade (19–22). This sequence of events includes: (i) single cancer cell detachment from the primary tumor and infiltration of the surrounding tissues (invasion); (ii) stimulation of neo vessel sprouting (neoangiogenesis); (iii) entering of cancer cells into blood vessels (intravasation), where these cells acquire the status of circulating tumor cells (CTCs); (iv) survival of CTCs to the hematogenous environment; (v) the leakage of CTCs from the bloodstream (extravasation) followed by their homing to distant organs, where they acquire the status of DCCs; and (*vi*) formation of micro metastatic bulks by DCCs and their adaptation to the new microenvironment (colonization) (23, 24). The metastatic cascade is full of rate-limiting steps, which explains why only a small percentage (0.02%) of DCCs successfully take root and rise into overt metastases (25). Indeed, after homing to a distant site, DCCs face a new microenvironment almost always devoid of growth permissive factors, resulting in DCC demise/senescence or entry into dormancy (1, 23, 26). As anticipated above, the acquisition of a dormant state is a strategy that enables cancer cells to perpetuate the disease while remaining under the radar for a protracted time, until both their fitness and the environmental conditions become permissive for growth (5). In this evolutionary process, the more DCC variants acquire genetic and epigenetic alterations, the higher is their probability of outgrowing in target organs.

Based on genetic comparative analysis studies, different evolutionary models have been proposed to explain the process of metastasization. In the linear progression model, metastases are late, even final events of primary tumor development (27) arising from the progressive accumulation of somatic alterations in cancer cells of the primary tumor (28, 29) that are under the selective pressure of heterotypic signals from the TME (30). Such a unidirectional timeline of events is initiated by the emergence of a cancer cell clone with metastatic capability followed by its dissemination to distinct organs. As a result, primary and metastatic sites are genetically related, although major differences can derive from the development of metastases from rare subclones (27, 31, 32) or the acquisition of specific genetic/epigenetic variation at the primary and/or colonization site. On the contrary, the parallel progression model assumes that DCCs develop early during tumor onset, perhaps even before the formation of overt primary lesions (33–38). This model implies that primary and metastatic tumors evolve independently from each other, resulting in them having a completely different genetic makeup (39, 40). Hence, cancer cells may constantly disseminate during primary tumor progression and evolve, giving rise to different cell variants, outside of the primary lesion. Finally, the tumor self-seeding model postulates a bidirectional exchange of cancer cells between parallel primary and metastatic lesions, denying the hypothesis of independent tumor evolution at primary and colonization sites (41).

Irrespective of the precise metastatic model, DCCs surviving this process are generally incompetent at growing in colonization sites and enter dormancy. This is clinically relevant, as beyond enhancing cancer cell fitness and aggressiveness, metastatic dormancy also induces resistance to therapy (5). Indeed, as conventional anticancer therapies target rapidly proliferating cancer cells, quiescence appears as the most consistent defense strategy of tumors to resist therapy. In particular, therapy-related dormancy preserves the survival of such cell subpopulations, which are the precursors of tumor relapse constituting the so-called minimal residual disease (MRD) (42) (Figure 1).

Cancer dormancy stands out as more than simple quiescence and clinical undetectability, instead, it is a multifaceted and plastic phenomenon with a tremendous impact on therapy outcome and patient survival. This is the reason why dormancy represents a major clinical conundrum and a hot research topic in oncology. We need to gain further insights into the mechanisms governing cancer dormancy and reawakening, as this would open new avenues for preventing or treating metastatic disease. To accomplish this need, a number of experimental preclinical and computational models have been developed.

## Models of Cancer Dormancy: Principles and Applications

Over the past two decades, an intensive wave of investigation in the field of tumor dormancy has led to the development of various experimental models that investigate the molecular mechanisms and circuitries regulating dormancy as well as the intricate cross-talk between dormant cancer cells and host immune cells (3, 43). Experimental strategies conceived to study cancer dormancy encompass: (i) *in vitro* and *ex vivo* models; (ii) *in vivo* models; (iii) mathematical and computational models. Table 1 summarizes these current methods, which are also briefly described here.

**Table 1:** Models for studying cancer dormancy.

***In vitro and ex vivo* models**	**References**
**2D cultures:**	
Cancer cells are cultivated on extracellular matrix (ECM) component-coated plates. Breast cancer + fibronectin + fibroblast growth factor-2	(44) (45)
**3D cultures:**	
Dormant cancer cells remain quiescent in 3D bioengineered models.	
**Biomaterial based model**	(46)
Breast Cancer + Basement Membrane Matrix Breast Cancer + Bone Marrow and Lung Niche Cells + laminin-rich ECM Breast Cancer + Bone Marrow Niche Cells + Collagen biomatrix Breast, Colon and Pancreatic Cancer + Stiff Col-Tgel Bladder, Prostate Cancer + Prostate Niche Cells + Amikagel Breast and Ovarian Cancer + Collagen gel Melanoma + Fibrin gel Brain Metastatic Breast Cancer + Hyaluronic Acid Hydrogel	(47) (48) (49) (50) (51) (52) (53) (54)
**Microfluidic based models/Organ-on-a-Chip**	
Breast Cancer + Hepatic Niche Cells + PEG hydrogel LiverChip and Breast Cancer Lung Cancer-on-a-Chip	(55–58)
**Bioreactor based model**	
Breast Cancer + Bone Niche Cells	(59, 60)
***In vivo*** **models**	
**Mouse vaccination and tumor challenge**	
BCL1 mouse lymphoma modelDA1-3b of acute myeloid leukemia	(61) (62)
**Experimental metastasis assays:**	
Cancer cells are injected directly into the circulation (e.g., tail vein, left cardiac ventricle, iliac artery)	(63) (64–66)
**Spontaneous metastasis assays:**	
Cancer cells are injected orthotopically or subcutaneously.	(67) (68, 69)
**Spontaneous tumor models:**	
Genetically engineered mouse models of oncogene ablation/induction (e.g., *Myc, Kras*) Transgenic mouse models (e.g., MMTV-PyMT, MMTV-HER2, RET)	(70–72) (33, 73)
**Resection mouse models**	(74, 75)
**PDX models**	(76–78)
**Mathematical and Computational models**	
**Ordinary differential equations**	(79–81)
**Mechanistic modeling**	(82, 83)
**Gene regulatory networks**	(84, 85)
**Systems biology models**	(86)

### *In vitro* and *ex vivo* Models of Cancer Dormancy

Despite constituting a highly simplified depiction of the TME, *in vitro* models of cancer dormancy provide major advantages including the unique possibility (i) to study, at a single cell resolution, the crosstalk between cancer cells and the other cellular and non-cellular components of the TME; and (ii) to functionally suppress or completely remove specific cell populations that are essential for animal survival and as such, difficult to be studied in *in vivo* models. The regulatory mechanisms identified through *in vitro* models, however, always need validation in more complex and realistic *in vivo* models.

Two-dimensional (2D) and three-dimensional (3D) cell cultures are the standard *in vitro* tools for investigating the mechanisms of cellular dormancy as well as the interactions with selected players of the microenvironment regulating major steps of dormancy such as cell cycle arrest, immunogenicity, differentiation, and therapeutic resistance. In the simplest 2D cell culture setting, cancer cells from either immortalized or primary cell lines are seeded on selected stromal components [e.g., fibronectin 1 (FN1), collagen I, collagen IV, among others] at clonogenic densities to favor cell interaction with the substratum and in the presence of microenvironmental soluble factors [e.g., epidermal growth factor (EGF) and basic fibroblast growth factor]. The effect of such extracellular matrix (ECM) factors on cancer cell dormancy, survival, and metastatic potential can then be evaluated by analyzing (as examples) cell clonogenic potential upon staining with crystal violet or cancer cell morphology, phenotype, cell cycle arrest, proteome and transcriptome employing standard methods of cellular and molecular biology (e.g., by microscopy, flow cytometry, western blot, qRT-PCR, and other techniques) (44, 45). In this setting, the 2D system can be easily perturbed by the addition of blocking antibodies, inhibitors, or peptides, partially mimicking the tumor microenvironmental conditions (44, 45). In this context, the recent development of microfluidic devices, bioreactors, and biomaterials, has driven researchers into a 3D cell culture-based multidisciplinary approach to detect, profile and even treat dormant cancer cells, spanning from fundamental biology to high-throughput screening (87–91). Indeed, cells cultured in a 3D model system more closely mimick the *in vivo* conditions and address most of the factors that can impact cancer dormancy, such as cell-to-cell and cell-to-ECM interactions, tissue architecture, proteomic and metabolomics profiles, and oxygen levels (92). 3D cell cultures can be generated by using either natural (Cultrex, laminin-rich ECM, collagen) (46–49) or synthetic biomaterials (collagen-based and fibrin-based hydrogels, amikagels, and hyaluronic acid hydrogels) (50–54). Moreover, organ-on-chip 3D models provide a way to study cancer dormancy at growing steps of complexity from a cell, to tissue till organ levels, and offer the possibility to perform a real-time, high-resolution analysis taking into consideration the inter-tissue interfaces, the fluid flows, and mechanical strengths, which are all features known to affect tumor dormancy (55–59). Similarly, bioreactors allow researchers to monitor and alter the chemical composition of the culture and thus to identify key chemical contributors to cancer dormancy and reawakening under controlled conditions (60).

Although highly informative and relatively simple, *in vitro* models are not devoid of caveats. The most significant hurdles of the *in vitro* systems are: (i) the need, in multicellular cultures, to optimize culturing protocols allowing the growth and survival of different cell types, (ii) the needs of organ-specific stromal cells, which are usually difficult to obtain, (iii) the difficulty of mimicking the dynamic evolution of the TME composition, and (iv) the challenge of replicating the complexity of the TME, and most notably the role of the immune system. Indeed, *in vivo* models represent a logical extension of *in vitro* findings providing a more comprehensive approach and enabling data validation.

### *In vivo* Models of Cancer Dormancy

Five broad approaches are currently employed to investigate cancer dormancy *in vivo*: (i) vaccination assays, (ii) metastasis assays, either in induced or spontaneous settings, (iii) spontaneous tumor models, (iv) resection mouse models, and (v) patient-derived xenograft (PDX) models.

In the vaccination assay, irradiated or otherwise killed malignant cells are inoculated into immunocompetent syngeneic mice. One-to-2 weeks later, the immunized animals are challenged with living cancer cells and monitored for the presence of persistent dormant cancer cells over long term follow up (from a few months to 1 year) (61, 62). As it stands, the gold-standard approach to evaluate the multi-organ dormancy of tagged cancer cells relies on metastasis assays. Metastases can be experimentally induced by injecting cancer cells into the tail vein (63–65) or the iliac artery (66). Otherwise, cancer cells can be injected subcutaneously or orthotopically and spontaneous metastatic potential can be monitored over time (67–69), or into genetically engineered mice that develop metastatic cancers (33, 70–73), or even humanized PDX models (76–78) can be used. All these assays allow *in vivo* live animal imaging and real-time monitoring of metastasis formation and growth, they provide countless insights into the mechanisms of metastatic dormancy and tumor persistence. Of note, as surgery could trigger metastatic relapse in patients with breast cancer (93), are so-called resection mouse models, which offer the possibility to link primary cancer surgery to the appearance of secondary disease at distant anatomical sites (74, 75) potentially helping unveil mechanisms of cancer cell dissemination and reawakening.

These multiplicities of *in vivo* models offer a holistic view of cancer dormancy and represent pre-clinical tools for clinical validation and intervention. However, *in vivo* studies also have some limitations. Indeed, cancer dormancy takes place over a long time frame and asynchronous heterogeneous dormant cancer cell populations are difficult to track. In this sense, the integration and merging of experimental data with mathematical models and computational simulations may provide insights and a better understanding of the regulatory circuits and the biological behaviors underlying dormancy, with invaluable benefits to translational research.

### Mathematical and Computational Models of Cancer Dormancy

The last 15 years have witnessed significant advances in mathematical modeling and computational simulations of complex biological processes such as cancer evolution, response to therapy, and even dissemination and dormancy. The use of mathematics in cancer research, known as mathematical oncology, encompasses knowledge-based differential equation models that simulate and predict tumor dynamics and response to therapy. Mathematical oncology offers insights into the complexity and multiscale nature of cancer cell dormancy and dissemination, (i) by integrating experimental and clinical information (79–81), (ii) by mechanistically modeling tumor evolution and progression as a functional consequence of the complex interaction between cancer cells and the surrounding TME (82, 83), and (iii) by predicting and simulating the molecular pathways involved (84, 85). More recently, systems biology, a multidisciplinary approach that integrates cancer research and medicine, genetics and epigenetics, mathematics, physics, and bioinformatics has gained momentum in the study of cancer dormancy and reawakening, as provides a more comprehensive view of the dynamics of these complex processes (86).

The optimization, application, and integration of all these models will help our understanding of the complexity of cancer dormancy and the multiscale nature of cancer progression. Undoubtedly, this is a promising path forward to validate and translate experimental findings in clinical settings and overcome therapeutic resistance in cancer.

## Cancer Dormancy and Cancer Stemness: Parallels and Differences Between Culprits of Relapse

CSCs are the subpopulation of stem-like cells within the tumor mass that possess unique stem-like features such as long-term self-renewal capability, multi-lineage differentiation, and high resistance to stress and apoptosis (9, 94). Based on these properties, CSCs are considered the seeds of tumor initiation, progression, and metastatic relapse and mainly responsible for therapy failure and poor clinical outcomes (9, 94). Historically presumed to be a very small and quiescent subpopulation, it is now clear that CSCs may not always adhere to this model. Indeed, recent evidence shows that CSCs can be relatively abundant (at least in some tumors), able to alternate between dormant and proliferating states, characterized by a high degree of heterogeneity and plasticity over space (i.e., in distinct tumor regions) and time (i.e., at distinct tumor progression stages) (9). Moreover, subsets of CSCs were reported to differentiate into heterogeneous lineages of cancer cells including non-stem cells, and *vice versa* differentiated cells to undergo cell dedifferentiation and even adopt CSC features (95, 96). CSCs reside in niches, which are specialized regions within the TME, preserving CSC survival and metastatic potential and regulating dormancy-reawakening switches (97). However, to date, a univocal definition of CSCs is still missing, and a unified model of genetic and phenotypic biomarkers is very difficult to achieve. In light of this evidence, resting CSC functional markers on the most threatening properties of CSCs may likely be the key.

One such property is the ability of CSCs to enter and exit from dormancy that, in the majority of cancer types, is the *sine qua non* condition for surviving therapy and initiating metastases, which are the two lethal features of CSCs. Based on this striking analogy, some investigations have proposed that CSCs and dormant cells are two sides of the same coin (6, 98). Indeed, ever-increasing data show the parallels at the molecular level, between dormant DCCs and CSCs. To give some examples, the activation of the p38 mitogen-activated protein kinase 1 (MAPK1) can induce dormancy in differentiated cancer cells (99) as well as in CSCs (100). Similarly, the induction of the mammalian target of the rapamycin (mTOR) signaling pathway could preserve both the survival of dormant DCCs (101) and the quiescence of CSCs (102).

Strengthening these findings, more recently, the activation of mTOR was able to enrich the pool of CSCs within DCCs in bone marrow (BM) metastatic niches in prostate cancer models, through a mechanism involving the release of growth arrest specific 6 (GAS6) by osteoblasts (103). Along with this, the Notch and Wingless (Wnt) pathways, which are essential for the maintenance of cancer stemness (104–106), were proven to promote cancer cell reawakening in different solid tumors (107, 108). Notably, these pathways could promote cell cycle progression in a fashion dependent on the protooncogene c-Myc, while their inactivation was associated with CSC senescence and tumor dormancy (109–112). Furthermore, c-Myc could trigger the polycomb repressor complex 1 component (PRC1) Bmi-1 expression, which in turn seems to correlate with breast cancer patient relapse years after treatment (113) and to influence the self-renewal capability of breast CSCs (114). Other examples proving the molecular similarity between dormant DCCs and CSCs include the interleukin 6 (IL-6) cytokine leukemia inhibitory factor (LIF)-LIF receptor (LIFR) axis, which appears to have a role in preserving both dormancy and cancer stemness, at least in the breast cancer setting (115). Autophagy, an evolutionarily conserved process through which cells survive metabolic stress conditions (116), can regulate the survival of dormant cancer cells and CSCs (117–120).

Finally, mechanical cues of the ECM and the epithelial-to-mesenchymal transition (EMT) process, may be functionally important for inducing stem traits in cancer cells and for promoting their metastatic outgrowth (121–123). For example, the Zinc Finger E-Box Binding Homeobox 1 (ZEB1), a key regulator of EMT, was shown to contribute to the cellular response to microenvironmental *stimuli*, such as local inflammation and the tumor promoter transforming growth factor-β (TGF-β), by activating a transcriptional program that pushes DCCs out of dormancy, committing them with stem-like features (124, 125). Similarly, the hypoxia-induced lysyl oxidase like-2 protein (LOXL2) can promote EMT and endow breast cancer cells with the ability to switch from dormant non-CSCs into proliferating metastatic CSCs (123). In this context, analyses in colorectal cancer models have recently revealed that the EMT-related factor ZEB2 coordinates a program of therapy resistance of quiescent cancer cells (126). Of note, these cells, which pre-exist in therapy-naïve tumors, show recognizable stem-like traits and behaviors (126). On the whole, these findings suggest again that the binomial dormant DCCs and CSCs could be interchangeable.

However, not all CSCs are dormant (9); and not all dormant cells are CSCs (127). Dormant cancer cells likely comprise both CSC and non-CSC subpopulations (7). Moreover, CSCs do not necessarily retain dormant-like features owing to their capacity to switch from dormant to proliferative states (128). Based on their tendency to enter dormancy, cancer (stem) cells can be broadly grouped into (i) dormancy-competent CSCs, (ii) dormancy-incompetent CSCs, (iii) cancer repopulating cells, and (iv) DCCs (7, 129). Dormancy-competent CSCs are endowed with the ability to switch between dormancy and reawaking states, a plasticity that fosters their metastatic potential and resistance to therapy (7, 129). Conversely, dormancy-incompetent CSCs are usually enriched in advanced diseases and are characterized by a loss in the ability to enter dormancy, possibly due to the progressive accumulation of somatic mutations in the mechanisms governing dormancy entry (7, 129).

Indeed, as the tumor progresses and the microenvironment evolves, CSCs accumulate epigenetic and genetic alterations despite their robust DNA damage response (130), and dormancy-competent CSCs may turn into dormancy-incompetent CSCs (129). Cancer-repopulating cells are the subset of CSCs able to self-renew post-therapy and thus responsible for relapse and metastatic onset (7, 129). Finally, DCCs, either with stem-like or differentiated features, lie in secondary distant organs and the bloodstream (in this latter case, acting as CTCs) and preserve the ability to reawaken and fuel metastatic outgrowth (7, 129).

As above described, striking parallels exist between dormant DCCs and dormant CSCs. These analogies also apply to the microenvironmental cues, encompassing biological, biochemical, and biophysical factors, that coordinate both DCC outgrowth and CSC self-renewal. Further research in this area could uncover new similarities that ultimately may offer therapeutic solutions for unmet medical needs.

## Microenvironmental Cues Cooperate to Tip the Balance Between Cancer Dormancy and Reawakening

The TME is a complex and dynamic ecosystem made up of a heterogeneous population of cancer cells and resident or infiltrating non-cancer cells [mainly leukocytes, including lymphocytes and tumor-associated macrophages (TAMs), cancer-associated fibroblasts, endothelial cells, and pericytes]. These are surrounded by the ECM and a mixture of secreted molecules encompassing lymphokines, cytokines, growth factors, and metabolites, among others. Cancer cell behavior and fate are profoundly influenced by the constant and evolving interplay with microenvironmental players, which often corrupt cancer cells to survive and eventually give rise to overt disease. The TME thus represents the background where physical and chemical perturbations tip the balance quiescence vs. proliferation. Quiescence and proliferation, in turn, come into sharp focus as by-products of the co-evolution of cancer cells and their microenvironments. Indeed, it is emerging that, in response to mitogenic and stress-signaling pathways, cancer cells trigger a set of complex intracellular molecular programs, thus underscoring a situation in which intrinsic mechanisms perfectly meet the cooperative action of extrinsic factors (3). Such intrinsic molecular pathways are beyond the scope of this review and have been extensively reviewed elsewhere (131–134). In this review, we will only cover the different microenvironmental cues governing dormancy regulation, with particular emphasis on CSCs and metastatic outgrowth.

### Cancer Niches: More Than Just Fertile Soils

Niches are specialized areas of the TME that regulate cancer (stem) cell fate and properties by the joint action of cell-cell and cell-ECM crosstalks and the messages delivered by paracrine factors.

***Metastatic niches*** are the fertile environments of secondary organs (i.e., BM, lymph nodes, lungs, liver, and brain) that provide favorable conditions for the seeding of DCCs with stem-like and non-stem-like features (135). Indeed, metastatic niches guarantee the nutrient and oxygen supply required for cell proliferation, thus setting the point for cancer (stem) cell proliferation or quiescence (135).

A body of evidence indicates that the BM frequently hosts DCCs derived from different primary organs, including breast, colon, prostate, head, and neck (136), although these DCCs rarely develop bone metastases (137). This observation suggests that BM metastatic niches could delay or even prevent tumor mass sprouting by inducing a state of dormancy (138), a situation observed in expanded hematopoietic stem cells (HSCs) undergoing differentiation (139). In line with this hypothesis, metastatic niches reportedly provide unique signals promoting quiescence and long-term survival. For example, Notch2, which is known to induce cancer cell proliferation in primary breast carcinomas (108), was recently shown to have an opposite effect in metastatic BM niches, favoring the quiescence and long-term survival of disseminated breast CSCs (140).

The Wnt pathway, which in its canonical form acts as a regulator of processes like cell proliferation and cell stemness (141), is also inversely associated with cancer cell dormancy (107, 142), was reported to induce dormancy of prostate cancer cells populating the BM niches, via a mechanism involving the non-canonical receptor tyrosine kinase-like orphan receptor 2 (ROR2)/Siah E3 Ubiquitin Protein Ligase 2 (SIAH2) signal, resulting in the inhibition of the canonical Wnt/β-catenin pathway (143). In this study, a negative correlation between ROR2 expression and metastasis-free survival in patients with prostate cancer was observed, potentially offering new potential therapeutic opportunities. These data are in line with previous observations of a role for non-canonical Wnt signaling in maintaining HSCs in a quiescent G_0_ state (144). At odds with this is the fact that canonical Wnt signaling, out of the BM, is generally inversely associated with cancer cell dormancy in different tumor types (107, 142). On the whole, these observations show opposite effects led by the same factors in different metastatic niches, where they likely face different microenvironmental factors. This further supports the hypothesis that HSC niches may host dormant cancer cells.

Other microenvironmental signals involved in dormancy at the metastatic site include TGF-β, bone morphogenetic proteins (BMPs), and LIFR. Firstly described as a potent inhibitor of HSC proliferation (145, 146), TGF-β is now recognized as another major factor that, once released by osteoblasts (one main BM stromal cell type), keeps DCCs and CSCs in a state of protracted dormancy (147, 148). This effect mainly relies on the triggering of the Gas6 receptor Axl (148) and the downstream activation of the p38 MAPK signaling (147). Similarly, the production of BMPs by BM stromal cells was associated with DCC hibernation. Specifically, the presence of BMP7 induced dormancy of prostate CSCs by activating the MAPK p38, and by fostering the expression of the cell cycle inhibitor p21 and the metastasis suppressor gene N-myc downstream-regulated gene 1 (NDRG1) (100). Accordingly, a variant of BMP7 (BMP7v) reportedly halted the metastatic spreading of colorectal CSCs by inhibiting the EMT program and by forcing cancer cell differentiation (149). In line with these observations, blocking BMP ligands via the TGF-β inhibitor Coco reawakened dormant breast CSCs and favored disease outgrowth in lung niches, which are known permissive soils (150). Notably, in a large cohort of patients, Coco-related metagenes predicted metastatic relapse in the lung, but not in the BM nor the brain, suggesting that Coco could be an organ-specific regulator (150). Finally, in breast cancer patients, low LIFR levels were shown to correlate with poor prognosis and with the appearance of overt metastasis along with the loss of CSC-associated genes (115). This is in line with previous observations which indicate that IL-6 plays a role in reawakening breast CSCs from therapy-induced dormancy (151).

Beyond reacting to soluble factors, DCCs also engage with other cell types of the metastatic niche, as well as with the ECM. Experimental studies show that breast cancer cells prime mesenchymal stem cells (MSCs) residing in BM niches to transfer microRNAs (miRNAs) via exosomes, which in turn promote cancer cell quiescence and drug resistance (152, 153). Apparently at odds with these observations, using a 3D co-culture model, Bartosh et al. demonstrated that DCCs from breast tumors cannibalize surrounding MSCs, resulting in an increased survival and tumor mass dormancy (154). Osteoblasts and osteoclasts, which are BM stromal cells with opposite physiological functions (155), also play opposite roles in the regulation of DCC dormancy. This was shown in myeloma DCCs, which entered dormancy while engaging with osteoblasts in the endosteum, while they started proliferating (i.e., reawakened) upon interaction with osteoclasts (77). Accordingly, as reported above, in a prostate cancer model, osteoblasts induce mTOR signaling by releasing GAS6, and this preserves CSC dormancy (103). Moreover, in breast cancer models, osteoclasts were recruited in the proximity of DCCs, supporting DCC growth into overt metastases (156). At the molecular level, cancer cell reawakening appears dependent on soluble receptor activator of nuclear factor- kappa B Ligand (sRANKL) signaling (77).

Accordingly, in breast cancer models, osteoclasts were found to be recruited in the proximity of DCCs and to support their growth into overt metastases (156). Along with this, a recent study in lung metastatic niches demonstrated that sustained inflammation and the interaction of DCCs with immune cells promote the formation of neutrophil extracellular traps (NETs, networks of neutrophil-derived extracellular fibers) in turn driving the switch from dormancy to reawakening (157). This effect was associated with the activity of neutrophil elastase and matrix metalloproteinase 9 (MMP9), two NET-associated proteases which sequentially remodel the ECM and activate the integrin α3β1 on cancer cells, eliciting downstream mitogenic signaling culminating in cellular dormancy.

The ***perivascular niche***, a tumor promoting milieu made up of a multitude of microvessels, regulates dormancy of cancer cells disseminated into BM, the lungs, and brain from various primary tumors (48, 158–160). Perivascular niches are characterized by the high availability of oxygen, nutrients, and paracrine factors, which renders them a permissive environment for the proliferation of DCCs and CSCs (161, 162). Accordingly, distinct types of CSCs and DCCs localize in the perivascular niches, growing in the proximity of capillaries (97, 163). It recently emerged that bidirectional interactions between these cells and components of the perivascular niche, including endothelial cells, are relevant for tumor evolution. The pool of glioblastoma CSCs residing at perivascular niches were shown to engage integrin α7-laminin interactions that foster invasiveness as well as self-renewal and growth potential (164), all features correlating with a dismal prognosis (165). Moreover, breast cancer cells that infiltrate lung metastatic niches induced the expression of the matricellular protein periostin (POSTN) in endothelial cells. In turn, POSTN contributed to CSC survival, nurturing micro to full macrometastases via a mechanism dependent on the activation of the Wnt signaling (166) and the activity of TGF-β1 (48). Other ECM components of the perivascular niche that influence metastasis include osteopontin and tenascin C (167–169). Emerging evidence indicates that these proteins act as primary regulators of CSC survival, self-renewal, and reawakening via the activation of transcriptional programs centered on Wnt, Nanog, and POU domain, class 5, transcription factor 1 (POU5F1, best known as Oct-4) (167–169).

Of note, is the fact that there is a certain degree of heterogeneity in endothelial cells of the perivascular niches. Thus, while endothelial cells of the sprouting neovasculature were shown to foster metastatic outgrowth, those of stable microvasculature mostly preserved and promoted cancer cell dormancy through the tumor suppressor thrombospondin-1, acting as a rate-limiting step for disease re-occurrence (48). Moreover, dormant and proliferating breast cancer cells displayed a distinct localization in perivascular areas (160). More precisely, dormant cells were shown to reside predominantly close to perisinusoidal venules expressing high levels of the inflammatory vascular cell adhesion molecule E-selectin, which favors the entry of cancer cells into the BM, and of the stromal cell-derived factor 1 (SDF-1), which anchors cells to the niche through its interaction with the C-X-C chemokine receptor type 4 (CXCR4), respectively (160).

### The ECM: A Biochemical and Biophysical Niche for Cancer Cells

The ECM, commonly defined as the non-cellular component of a tissue, is a highly dynamic and physiologically active structure, that provides biochemical and biophysical support for surrounding cellular components (170). Characterized by a continuous remodeling over space and time, the ECM also represents a biological barrier, an anchorage site, and a movement track, playing major roles in regulating cellular interactions and communications (170). The ECM is tightly organized during embryogenesis and tissue homeostasis, but becomes extremely deregulated and deranged in cancer (171).

Emerging evidence suggests that the ECM may serve as a niche for DCCs and CSCs, influencing cell survival and proliferation, and thus dormancy (171, 172). Thus, downregulation of the urokinase plasminogen activator receptor (uPAR), which is involved in cell/ECM interactions, affected the capability of head and neck squamous cell carcinoma cells to interact with integrins, in turn causing deactivation of mitogenic pathways and induction of dormancy (173). Along with this, tissue stiffness (a mechanical property of the TME) and its underlying mechanotransduction pathways are also involved in tumor progression and metastasis (122, 174). Thus, in breast cancer models, the crosslink between fibrosis-associated deposition of type I collagen and integrin β1 or lysyl oxidase (LOX), was described to create a growth-permissive microenvironment capable of reawakening DCCs, thus supporting proliferative metastatic growth (46, 175). This occurred through the activation, downstream of integrin β1, of players including focal adhesion kinase (FAK), non-receptor tyrosine kinase (Src), ERK, and myosin light chain kinase (MLCK) (46). In this context, there is interesting evidence that pharmacological co-inhibition of Src and MEK1/2 prevented disease recurrence by killing dormant breast and ovarian DCCs (176, 177). Similarly, interstitial collagen I was described to favor the interaction between the tetraspanin Transmembrane 4 L Six Family Member 1 (TM4SF1) and the collagen receptor tyrosine kinase Discoidin domain receptor family, member 1 (DDR1). This led to the expression of the stem related factors SRY (sex determining region Y)-box 2 (SOX2) and NANOG, driving multiorgan metastatic reactivation in the lung, bone, and brain (178).

The dormant-to-proliferative metastatic switch is also favored by a global reconfiguration of the cytoskeletal architecture of DCCs often mediated by the integrin β1 signaling. Thus, using a model of lung disseminated breast cancer cells, Green et al., demonstrated that, cancer cells respond to integrin β1-mediated fibronectin production and signaling by activating MLCK, resulting in the generation of actin stress fibers and entry into a proliferative state (179). The ability of integrin β1 signaling to promote cell-cycle progression seems also to rely on FAK activation (180). In particular, Weinberg's team showed that soon after extravasation into the lungs, breast cancer cells arrest their proliferation due to their inability to engage stable adhesions with ECM components. Later on, some cancer cells acquiring an elongated morphology developed abundant cell-matrix adhesion plaques, which in turn triggered the integrin β1-FAK signaling and promoted exit from dormancy (180). Src family kinases (SFKs) also act downstream the integrin-triggered dormant-to-proliferative switch (176). Moreover, using an *in vitro* model of stiff-soft tunable matrix it was revealed that fibrosis related integrin β1 and FAK signaling increased mitogenic *stimuli* by inducing protein kinase B (PKB/Akt) and signal transducer and activator of transcription 3 (STAT3). In this setting, only cells grown in soft matrix supports expressed CSC markers (181), suggesting that a pliable microenvironment might support cancer cell stemness, a hypothesis that is intriguing but which still requires *in vivo* validation. Finally, the association between matrix stiffness and cancer cell proliferation appears to be influenced by endothelial cells (182). More precisely, in a stiff environment, endothelial cells express the matricellular protein cysteine-rich angiogenic inducer 61 (CYR61), which in turn induces a β-catenin-dependent upregulation of N-cadherin levels. This lets cancer cells stably interact with the endothelium and thus enter the bloodstream and metastasize (182).

To add further layers of complexity, a recent study demonstrated that a stiff matrix could also induce dormancy (183). In this study, cancer-repopulating cells, when coping with a harsh environment, activate an epigenetic program that leads to the transcription of ten-eleven translocation 2 (Tet2) hydroxymethylating enzyme. Tet2, in turn, activates the cell cycle suppressors p21–p27 and induces integrin β3 downregulation, respectively, promoting and preserving dormancy (183). Moreover, a recent deep single cell analysis revealed a high phenotypic heterogeneity in dormant cancer cells, encompassing pools of quiescent, senescent, and actively proliferating cells (184). The characterization of cells entering long-term dormancy demonstrated that these cells adhere stably to a stiff matrix through integrin α5β1 and rho-associated kinase (ROCK)–mediated cell tension. Moreover, the capability to exit from dormancy appears strictly connected to the ability to trigger MMP-mediated FN1 degradation (184).

**Figure 2:**
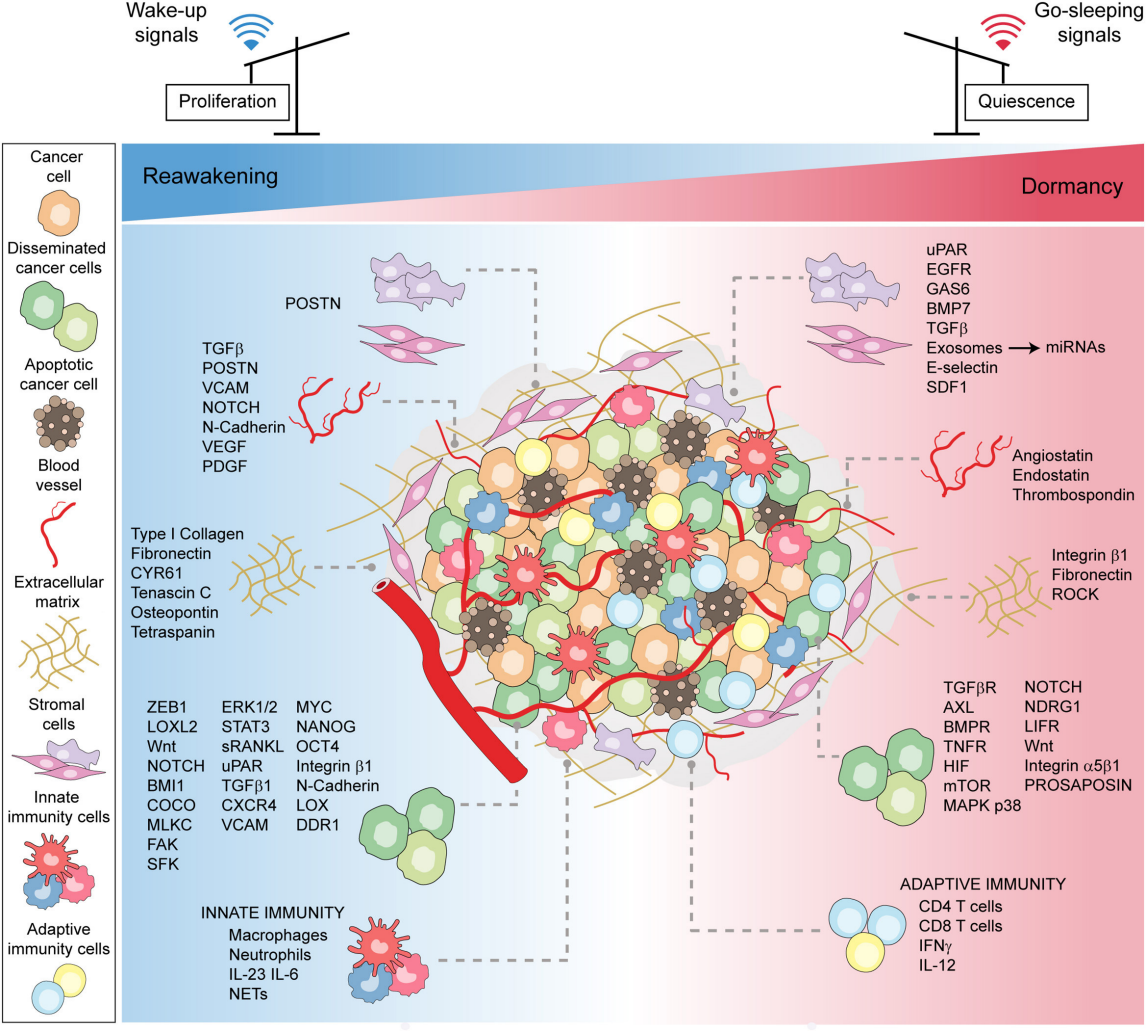
Microenvironmental patterns tuning cancer dormancy, reawakening, and stemness. A schematic model showing the plethora of microenvironmental cues, encompassing the cellular, molecular, and physical factors, that converge to induce either stress-related or mitogenic signals to cancer cells. The bulk of cancer cells, encompassing disseminated and stem cells, in the face of contextual signals, either enter or exit dormancy.

In conclusion, disseminated cancer (stem) cells and their environment engage in an intricate molecular cross-talk, regulating the entry into and the exit from dormancy and thus determining cancer cell fate (Figure 2).

## Angiogenic Switch and Angiogenic Dormancy

A hallmark of progressive cancer growth, in both primary and secondary tumors, is the induction of tumor vasculature, a process termed the “angiogenic switch” (185, 186). Indeed, like healthy tissues, tumors need both an appropriate supply of oxygen/nutrients and a way to remove waste products (187). However, unlike physiological angiogenesis, in which new vessel sprouting is a highly regulated and self-limited process, tumor angiogenesis lacks growth controls resulting in continuous and deregulated vessel production (185). This leads to a structurally and functionally abnormal tumor vascular network characterized by new vessels with dead ends, which results in low oxygen tension (hypoxia), the paucity of metabolites, and imbalanced expression of angiogenic factors. This latter eventually stimulates further abnormal angiogenesis (185). As neovascular supply is crucial for tumor growth, cancer cells, including those integrated into the vessel walls (188), undergo adaptive dormancy, also known as angiogenic dormancy (186, 189). During angiogenic dormancy, cancer cell proliferation rate is balanced by enhanced apoptosis induction. This equilibrium maintains tumors that are microscopic and undetectable, for extended times (12).

Currently, there are three subtypes of hypoxia and related cancer cell adaptive mechanisms (190). First, acute hypoxia is characterized by transient perturbation in perfusion lasting from a few minutes up to a few days. Reportedly, cancer cells facing acute hypoxia decrease oxidative metabolism and activate autophagy, yet retaining high proliferative potential (191–193). Second, chronic hypoxia is mainly related to the presence of abnormal neo-vessels, leading to limited perfusion and oxygen supply. This long-lasting phenomenon is linked to a state in which cancer cells remain persistently dormant (192). Finally, cycling hypoxia is characterized by oxygen fluctuations in parallel with intermittent phases of cancer cell dormancy and reawakening that have been associated with increased tumor aggression (190, 194).

The balance between the angiogenic switch and angiogenic dormancy is a finely-tuned process regulated by integrated microenvironmental factors, including the pro-angiogenic vascular endothelial growth factor (VEGF), platelet-derived growth factor (PDGF), anti-angiogenic thrombospondin-1, angiostatin, and endostatin (189). Prosaposin has been described as another regulator of metastatic growth arrest (195). Once produced by cancer cells, prosaposin acts in a paracrine and endocrine fashion inducing the expression of thrombospondin-1 in stromal cells at primary and distant tumor sites, which blocks neoangiogenesis and delays tumor growth (195). Many niche components also play a role in regulating angiogenesis. Indeed, CSCs, seem able to transdifferentiate and directly contribute to the formation of abnormal vessels, thus supplying for the absence of true angiogenesis (196, 197). Moreover, CSCs often promote a considerable enhancement of VEGF levels, both by a direct production and by stimulating a pro-angiogenic activity in stromal cells localized in the proximity of the niche (198–200). Along with this, some stem-related factors such as Notch also act as angiogenesis promoters (201, 202), while anti-angiogenic factors (i.e., thrombospondin-1) are associated with inactivation of the stem-related transcriptional factors (i.e., MYC) (203), which in turn promote dormancy (109). Of note is the fact that CSCs adopt further adaptive mechanisms to cope with hypoxia, among which the expression of the hypoxia-inducible factors (HIFs) and HIF-regulated genes (204) that induce cellular dormancy by activating p21 signaling (205). In a seminal work, Almog et al. characterized a transcriptional rewiring of cancer cells undergoing an angiogenic switch (206). This switch was associated with downregulation of the angiogenesis inhibitor thrombospondin and upregulation of genes not hitherto linked to tumor dormancy, such as endothelial cell-specific molecule 1 (ESM1), 5'-ectonucleotidase, tissue inhibitor of metalloproteinase 3 (TIMP3), epidermal growth factor receptor (EGFR), insulin-like growth factor receptor (IGF1R), phosphatidylinositol 3-kinase (PI3K) signaling, Eph receptor A5 (EphA5), and histone H2BK (206).

In summary, these myriad microenvironmental components and their reciprocal interactions, represent the major culprits governing cancer (stem) cell dormancy and outgrowth, and are a clear index of the complexity of this regulation, offering additional potential targets for therapeutic intervention.

## Cancer (Stem) Cell Dormancy and Immunity: Productive Dialogs and Reciprocal Regulations

### Immune-Induced Dormancy: the Equilibrium Phase of Cancer Immunoediting

Over the past two decades, understanding of tumor biology has increased and revealed that the host immune system plays a dual role in cancer: it may both constrain and paradoxically aid tumor outgrowth. This phenomenon, which has been referred to as cancer immunoediting, passes through three phases, namely elimination, equilibrium, and escape (207).

During the elimination phase or immunosurveillance, cancer cells that escaped intrinsic control are destroyed by extrinsic, immune-mediated tumor suppressor mechanisms (208). The successful completion of this phase ensures cancer cell clearance and prevents the onset of the clinically apparent disease. However, rare cancer subclones may survive and progress into a phase of equilibrium, during which the immune system, by inducing a functional state of dormancy, might contain but not fully extinguish cancer cell growth. Of note, is the strong and relentless pressure exerted by the immune system during this phase, which may either control the outgrowth of occult tumors throughout the life of the host or sculpt less immunogenic variants that ultimately evade immune attack (208). Such immunoedited cancer cells, that are no longer susceptible to immune control, progress in the escape phase, emerging into clinically visible tumors (208). Of the three phases of cancer immunoediting, equilibrium is probably the longest and the most difficult to characterize. Clinical evidence on the existence of an equilibrium or tumor-dormancy phase came from the unintentional transmission of cancer from transplant organ donors to immunosuppressed recipients. In these cases, donors either were in durable clinical remission (209–211) or had no known history of malignancy (212, 213). Notably, cases of the rapid outgrowth of occult metastases were even reported when donors had glioblastoma, which usually does not metastasize (214). Similarly, metastatic recurrence of primary renal cell carcinoma soon after the post-transplant immunosuppressive medication was reported (215). These observations suggest a mechanism of immune-mediated control for occult malignancies and a progressive outgrowth of cancer cells under pharmacologically-induced immunosuppression, a condition required to prevent the recipient's rejection of the organ. The median time frame between transplantation and metastasis detection is relatively short, ranging between 3 and 36 months, with no differences between cancer types and the organ transplanted (211). As metastases generally take 6 to 12 years to emerge (35), it is plausible that under immune suppression, adaptive immunity cannot hold dormant cells in check, which thus exit from the equilibrium/dormant/persistent state (13). In line with these observations, in a variety of human tumors, it was reported that a 20 to 50 year interval from initial carcinogen exposure to the clinical detection of disease. Moreover, epidemiologic studies in autopsies revealed that microscopic foci of disease frequencies considerably exceed clinical incidence rates in various cancer types (e.g., thyroid cancer, prostate and breast carcinoma) (216–218). This gets stronger during the theoretical existence of periods of subclinical dormancy during tumor progression (219). However, none of these reports visualized tumor dormancy *de facto*, and they did not describe the immune effectors involved. Admittedly, clinical cancer dormancy is still poorly characterized and the role of innate and adaptive immunity in initiating and then stabilizing the dormant state is a matter of debate (220). However, we have strong evidence supporting the existence of an equilibrium phase governing clinical cancer dormancy. Indeed, tumors may chronically persist without symptoms for years and even decades before recurring either locally or at distant metastatic sites (4, 25, 221). Moreover, late relapses are relatively frequent in breast and prostate carcinoma patients after radical surgery (222, 223), in melanoma, thyroid and renal cell carcinoma (224, 225), non-Hodgkin's lymphoma (226), and acute myeloid leukemia (227).

In parallel, clinical and experimental studies have provided evidence that cancer cells can disseminate during premalignant stages of the disease, thus entering a protracted period of metastatic dormancy into target organs (35). Early preclinical suggestions of the capability of the immune system to hold cancer cells in a dormant/equilibrium phase were provided by transplant experiments in which immunodeficient mice adoptively transferred with T cells and then challenged with the murine B lymphoma BCL1 cells, were endowed with the capability to induce and maintain a state of tumor latency (228). Similarly, BCL1 dormant cancer cells resident in the spleen of immunized mice showed no evidence of disease 250 days after tumor rechallenge (229). In line with these findings, the adoptive cell transfer of tumor specific lymphocytes provided long-term protection from tumor development, retaining minor foci of dormant cancer cells on mouse models of prostate cancer (230) and lymphomas (231). Additional studies with mouse models of skin malignancies confirmed that the immune system may induce long-term latency of occult primary and metastatic carcinomas (232, 233). These findings are consistent with a role for anti-tumor immunity, and in particular T cells, in the maintenance of an equilibrium dormant state preventing tumor-cell growth. Pivotal studies from Schreiber's lab have further provided evidence and unveiled mechanisms of immune mediated dormancy. It was observed that the treatment of mice with low-dose methylcholanthrene (MCA) was followed by the development of aggressive tumors in only a few animals, with a sizeable percentage of the surviving mice free of disease. Deceptively, however, these mice bear dormant tumors that were held in check by the immune system. Indeed, when animals were treated with antibodies blocking T lymphocytes or neutralizing the cytokines IL-12 or interferon-γ (IFN-γ), tumors were released from immune control and outgrew (13). These findings validate previous observations of dormancy induced by CD8 T cell derived factors (228, 234). Moreover, MCA-induced sarcomas from immunodeficient mice were more immunogenic than those arising in immunocompetent hosts (235). Follow-up studies showed opposing, complementary roles for ILs, during the equilibrium phase. Specifically, IL-23 seemed to promote the survival and outgrowth of occult cancer cells, while IL-12 seemed to favor dormancy and thus prevent immune escape (236). At odds with previous reports, innate immune signaling is associated with the awakening of dormant cancer cells. Local inflammation in the lungs was shown to ignite the exit of DCCs from latency, and thus the growth into overt metastases through the activation of a previously silent EMT transition program (125). This provided a newfound knowledge of the dual role of the immune system in protecting the host against tumor outgrowth and in sculpting the immunogenic profile of evolving tumors, finally rendering them more fit to survive and progress in an immunocompetent environment (235).

On the whole, these observations suggest that immunity can maintain cancer cells in a transient dormant state, which as a matter of course, end in either tumor elimination or tumor escape. CSCs may play pivotal roles in preserving the cancer dormant state. Indeed, they cope with robust anticancer immune responses by subverting immune effector functions and by drastically reducing their visibility (237). At the same time, however, such an immunopriviledge may foster immune escape and cancer outgrowth (238). It remains to be elucidated whether immune-mediated dormancy is either always a matter of a bulk tumor or may also resemble cellular dormancy. Mining the mechanisms regulating immune-mediated tumor equilibrium will help solve this question, and will open the possibility of uncovering predictive signatures with invaluable prognostic and therapeutic implications.

### Dormancy as a Mechanism of Immune Escape: Sleeping in the Name of Survival

Immune escape is central to tumor persistence and relapse. Dormant cancer (stem) cells constitute the most critical, yet heterogeneous fraction of malignant cells able to evade host antitumor immunity (6, 7). Effective mechanisms of escaping immune control are (i) prevention of immune detection, (ii) prevention of immune activation, and (iii) activation of immune suppression (239, 240).

The immunogenicity of a tumor relies on a combination of antigenicity, i.e., the expression and presentation of tumor-associated antigens, and adjuvanticity, i.e., the release of alarmins and damage signaling (241). Cancer cells defective in either antigen presentation or production of adjuvant-like signals (or both) remain relatively invisible to the immune system and escape immune detection. The capability of dormant cancer cells to evade immune surveillance by reducing antigenicity has been reported (242, 243) and more recently confirmed through clinical immunogenomics (244, 245). Downregulation of the major histocompatibility complex class I (MHC-I) was ostensibly observed in quiescent cancer cells and CSCs isolated from different cancer types (238, 246). In a model of liver disseminated pancreatic cancer, dormancy-related loss of MHC-I was attributable to unresolved endoplasmic reticulum stress, and was responsible for hiding and protecting DCCs from T cell-mediated surveillance (247). Interestingly, the observation that, in hair follicles, Lgr5-GFP stem cells survive the adoptive transfer with antiGFP T cells by persisting in a dormant state, and reducing the expression of MHC-I molecules (248), further confirms that loss of antigen presentation is a common mechanism in quiescent cells, which CSCs adopt to escape immune attack. If the tumor does not manage to escape detection, then it can evolve to prevent the activation of a robust anticancer immune response. The immunosuppressive effects of cancer cells are mediated by (i) the secretion of soluble factors, (ii) the expression of inhibitory molecules, and (iii) the turning of infiltrating leukocytes into tolerogenic cells that, in turn, can suppress other tumor-specific immune cells. In a model of acute myeloid leukemia, the expression by cancer cells of the immune checkpoints CD274 (best known as PD-L1) and CD80 (also known as B7.1) prevented T cell activity and preserved cancer dormancy (62). Furthermore, the microenvironment itself can help quiescent cells elude immune control. Indeed, within the perivascular niche, the activity of effector T cells can be inhibited through the release of immune suppressive cytokines (such as IL-6) and the activation of the programmed cell death 1 (PDCD1, best known as PD1)-PD-L1 axis (249–251). In addition, tumor evolution seems to select for cancer cell clones resistant to the death effector mechanisms of the immune system. We recently discussed the genetic inactivation of the oncosuppressor caspase 8 (CASP8) and the death receptor FAS as strategic mechanisms cancer cells may adopt to evade apoptosis-mediated eradication by immune cells, mainly T and natural killer (NK) cells (5). These reports are in line with previous observations of dormant cancer cell-mediated escape from T cell induced apoptosis through deregulation of the suppressor of cytokine signaling 1 (SOCS1) cascade and overexpression of the pro-tumorigenic cytokine IL-3 (252).

Cancer cells defective for MHC-I molecules are optimal targets for NK cells, in which activation is MHC-unrestricted (253, 254). Evidence of evasion from NK mediated immunosurveillance by quiescent disseminated CSCs firstly came from Massague's lab. This team showed that by overexpressing the WNT inhibitor Dickkopf-related protein 1 (DKK1), CSCs enter a self-imposed quiescent state and downregulate the expression of UL16 binding protein (ULBP) ligands for NK cells, thus evading innate immunity and remaining latent in the long-term (255).

Additionally, dormant cancer (stem) cells may enter immune protected niches (also called immune-privileged niches), where they lie quiescent for extended periods (256). The capability of dormant niches to protect (cancer) stem cells from immune control is mainly due to the recruitment of regulatory immune cells, encompassing regulatory T (T_REG_) cells, myeloid-derived suppressor cells (MDSCs), and immunosuppressive TAMs and neutrophils (TANs) (257, 258). In particular, TAMs are recruited by diverse chemotactic factors—including tumor-derived colony-stimulating factor 1 (CSF1), vascular endothelial growth factor A (VEGFA), semaphorin 3A, CC-chemokine ligand 2 (CCL2), and CXC-chemokine ligand 12 (CXCL12)—and nullify the cytotoxic activity of CD8^+^ T cells by expressing the immune checkpoints PDL1 and B7-H4 (259, 260).

In addition, TAMs and regulatory dendritic cells can recruit T_REG_ cells and MDSCs, and foster their expansion and immunosuppressive functions (261–263). In brain metastatic loci, reactive astrocytes prevent CD8 T cell activation and recruit TAMs through the signal transducer and activator of transcription 3 (STAT3) activation program (264). Similarly, once expanded and polarized under gamma delta (γδ) T cell control (265), TANs act as pro-tumorigenic players in particular in metastatic niches, conferring highly immunosuppressive properties to the TME through the release of leukotrienes (266).

Finally, some tumors seem to evolve and acquire the capability to corrupt and turn immune effectors against themselves, thereby causing immune cell death through mechanisms that physiologically limit the antitumor immune response (240, 267, 268). These immune escape mechanisms can act in combination and make the tumor a formidable foe for the immune system, ultimately fostering the neoplastic outgrowth. Current integrated and single-cell based approaches that have been adopted to mine the immunome of primary and metastatic tumors seem extremely powerful, and may offer data that will soon implement the list of factors, cells, and mechanisms involved in immune escape.

### Clinical Detection of Dormant DCCS and CTCS

The identification and possible targeting of dormant DCCs which persist during MRD is of utmost importance to prevent disease recurrence. However, the clinical detection (and monitoring) of cancer dormancy is a challenge, making it difficult to validate the cancer dormancy model in patients. Indeed, per definition, cancer dormancy is a controlled chronic disease that persists without any symptom or sign until its underlying equilibrium is disturbed and local or systemic relapse occurs. Two major obstacles need to be overcome for the clinical detection of cancer dormancy. First, micrometastatic, dormant DCCs are almost undetectable using conventional high resolution, whole-body imaging tools. Second, the entire process involves a long time frame of disease latency. In the last two decades, a flurry of research efforts have focused on the identification and standardization of highly sensitive and specific assays to identify and characterize occult micrometastatic cancer cells, in particular, DCCs in BM aspirates, and CTCs in peripheral blood.

### The Current State of DCC and CTC Detection

Three methods are commonly used to detect and quantify DCCs and CTCs in liquid biopsies: (i) immunocytochemistry (IHC)/immunofluorescence (IF) staining followed by bright field/fluorescence microscopy; (ii) multicolor flow cytometry (MFC); and (iii) real time-polymerase chain reaction (RT-PCR). In this context, IHC and IF are the most widely used approaches as they provide the major advantage of evaluating and characterizing morphological criteria at a single-cell level (269, 270). On the contrary, MFC analyses are largely used to analyze biopsies from advanced stage metastatic cancer patients as they allow the rapid screening of tens of thousands of cells per second coupled with the possibility of isolating pure, viable cell subsets for further experimentation. As examples, isolated cells can be expanded either *in vitro*, by establishing primary cell cultures, or *in vivo*, by using xenograft models, and then used for functional analyses (271). A major drawback of these antibody-based technologies is the possibility of false positives, due to an “illicit” expression of markers in non-malignant cells—which can be the result of inflammation or injury (272), or even of the formation of chimeras by the fusion of cancer cells with immune cells (273)—and false negatives, due to the loss of marker expression (270). Finally, RT-PCR-based transcriptome analyses allow for the simultaneous and high sensitive detection of multiple factors, although the probability of false positive results due to contamination and amplification of transcripts from non-cancer cells is high. Besides, the presence of degrading enzymes could also give rise to false negative results (274). In these experimental settings, as DCCs and CTCs are a few tens dispersed in millions-to-billions of hematopoietic cells per milliliter of BM aspirate or blood, prior enrichment approaches through density gradient centrifugation and/or immunomagnetic bead separation are mandatory (269).

### Markers of DCC and CTC Detection, Isolation, and Characterization

As hematopoietic cells circulating in the peripheral blood and residing in the BM are mainly of mesenchymal origin, epithelial cancer cells from different solid tumors can be identified through epithelium-specific antigens such as (i) cytoskeletal-associated cytokeratins (CKs, in particular CK 8, 18, 19, and 20) (275, 276), (ii) surface adhesion molecules, such as the epithelial cell-adhesion molecule (Ep-CAM) (269), and (iii) growth factor receptors, such as the erb-b2 receptor tyrosine kinase 2 (ERBB2, best known as HER2) for breast cancer and the epidermal growth factor receptor (EGFR) for lung cancer. Moreover, to disseminate in distant anatomical sites, cancer cells lose cell-to-cell adhesion molecules and enter the EMT process. Therefore, markers of EMT, such as vimentin, FN1, twist family bHLH transcription factor 1 (TWIST1), snail family transcriptional repressor 1 (SNAI1) and 2 (SNAI2, best known as SLUG) can be used to detect cancer dormancy (277, 278). As described above, DCCs can show stem cell features (7), such as the expression of cell surface adhesion receptor CD44, the cell surface CD24, prominin (best known as CD133), and CD49 antigens, and the functional marker aldehyde dehydrogenase 1 family member A1 (ALDH1) (279). Notably, co-staining with specific markers helps discriminate between quiescent and actively proliferating DCCs and CTCs. The most common dormancy-specific markers are the lack of the nuclear antigen Ki67, and the expression of the nuclear receptor subfamily 2 group F member 1 (NR2F1), the basic helix-loop-helix family member e41 (BHLHE41, also known as DEC2), and the cyclin dependent kinase inhibitor 1 B (CDKN1B, best known as p27) (280). Because dormant cells activate cytoprotective programs (i.e., the UPR) to cope with environmental stresses, including hypoxia and glucose starvation, the expression of UPR proteins, such as the heat shock protein family A (Hsp70) member (HSPA5, best known as Grp78), can be analyzed (281).

### Major Limitations of DCC and CTC Detection and Future Directions

Despite the successful detection and enumeration of DCCs and CTCs, and the unceasing development of automated and high sensitive analytical methodologies (e.g., CellSearch, ImageStream, FAST, Epic, CytoTrack, and EPISPOT platforms) (270, 282, 283), achieving high yield and high purity remains a major challenge. Moreover, the high variability of the results is due to multiple reasons, including the heterogeneity of marker expression, the difficulties to recover intact and live cells, the bias of false positive and false negative data, and the lack of standardized protocols. This has prevented the implementation of DCC and CTC usage into the routine clinical practice (284–288).

Currently, next-generation sequencing (NGS) multi-“omics” technologies are providing large-scale data and more comprehensive characterization of the intricate molecular mechanisms underlying the hallmarks of cancer (289). The in-depth knowledge of disease development, treatment resistance, and recurrence risk facilitated by this will be fundamental in guiding treatment decisions. Very recent advances in single-cell analyses have enabled researchers to characterize intra-tumor heterogeneity (i.e., the heterogeneity among the cancer cells of a single patient, at the spatial or temporal level), identify rare cell subsets, and measure the mutational landscapes of different cancer cell populations, and thus guide diagnosis and treatment. However, mainly due to the prohibitive costs (single-cell), multi-omics analyses have not yet been implemented in the clinical setting, preventing the advancement of precision medicine. As there is a widely recognized need to detect and characterize dormant DCCs and CTCs in more detail, there will undoubtedly be a rapid development of new, standardized, and exploitable technologies in the near future, that will expedite DCC and CTC implementation in clinical settings to prevent relapse and thus improve outcome.

## Implications for Therapy

After years of bench studies on cancer dormancy, discoveries of the mechanisms regulating dormancy and reawakening could provide an opportunity for bedside translation. There are essentially two clinical options to target dormant cancer (stem) cells: (i) forcing them out of quiescence, so-called “lock-out” approaches, or (ii) sustaining their perpetual dormancy, so-called “lock-in” strategies (138, 290). Clinical trials were launched to study the safety and efficacy of both strategies (Table 2). Nonetheless, these strategies require detailed knowledge of the mechanisms underlying dormancy and tumor evolution, a clear view of which mechanisms are tissue-specific or instead common and thus universally exploitable, and the possibility/ability to stratify patients and distinguish those who could benefit from therapies targeting dormancy and those who could not.

**Table 2:** Clinical trials targeting the dormancy window in cancer patients.

**Description**	**ClinicalTrials.gov identifier**	**Drug(s)**	**Number of patients**	**Recruitment status**	**Phase**	**Results**
Pilot study to evaluate the impact of Denosumab on DTCs in patients with early stage breast cancer	NCT01545648	Denosumab	4	Terminated (low accrual)	2	N/A
Pilot study of mobilization and treatment of DTCs in men with metastatic prostate cancer	NCT02478125	Burixafor hydrobromide, G-CSF, Docetaxel, or in combination	3	Terminated (low accrual)	1	N/A
Effect of Trastuzumab on DFS in early stage HER2-negative breast cancer patients with ERBB2 expressing DTCs	NCT01779050	Trastuzumab	7	Active, not recruiting	2	All patients experienced eradication of HER2/neu-positive ITCs from bone marrow; reduction in the number of ITC-positive patients
Zoledronic acid in the treatment of breast cancer with minimal residual disease in the bone marrow (MRD-1)	NCT00172068	Zoledronic acid in combination with calcium/vitamin D	96	Terminated	2	All patients treated became DTC negative; untreated patients 12 months after diagnosis had significantly shorter OS
Secondary adjuvant treatment for patients with ITCs in bone marrow	NCT00248703	Docetaxel	1,028	Active, not recruiting	2	79% of patients became DTC negative; enhanced metastasis-free survival in patients with DTC elimination
Gedatolisib, Hydroxychloroquine or the combination for prevention of recurrent breast cancer (GLACIER)	NCT03400254	Hydroxychloroquine, Gedatolisib, or combination	0	Withdrawn	3	N/A
Phase II pilot trial of Hydroxychloroquine, EVErolimus or the combination for prevention of recurrent breast cancer (CLEVER)	NCT03032406	Hydroxycholorquine, everolimus, or combination	60	Recruiting	2	N/A
Prolonged Tamoxifen compared with shorter Tamoxifen in treating patients who have breast cancer	NCT00003016	Tamoxifen citrate	20,000	Terminated	N/A	N/A
Pilot study of 5-Azacitidine and All-trans retinoic acid for prostate cancer with PSA-only recurrence after local treatment	NCT03572387	Combination of 5-Azacitidine and All-trans retinoic acid, or no treatment	20	Recruiting	2	N/A
Phase II study comparing chemotherapy in combination with OGX-427 or placebo in patients with bladder cancer	NCT01454089	Gemcitabine and Cisplatin in combination with OGX-427	183	Completed	2	N/A
OGX-427 in castration resistant prostate cancer patients	NCT01120470	OGX-427 and prednisone in combination	74	Completed	2	N/A
Safety and efficacy of ABT-510 in subjects with advanced renal cell carcinoma	NCT00073125	ABT-510/Thrombospondin-1 mimetic	103	Completed	2	N/A
PROvenge treatment and early cancer treatment	NCT00779402	Sipuleucel-T	176	Completed	3	N/A
Sunitinib malate or Sorafenib tosylate in treating patients with kidney cancer that was removed by surgery	NCT00326898	Sunitinib malate or sorafenib tosylate	1,943	Completed	3	None of patients treated showed survival benefit relative to placebo

*DC, dendritic cell; DFS, disease free survival; DTC, disseminating tumor cell; ITC, isolating tumor cell; N/A, not applicable; OS, overall survival*.

As dormancy represents a mechanism by which cancer cells evade current conventional antiproliferative therapies, ***lock-out*** strategies aim at reawakening and forcing dormant cells into proliferation before treatment. According to this principle, exit from dormancy wakes up cancer cell sensitivity to conventional chemo and radiation therapy as well as some types of target therapy. Inhibitors of Polo-like kinase1 (Plk1), for instance, appear highly effective against proliferating colorectal CSCs (128). Notably, dormant CSCs survive the treatment with Plk1 inhibitors but retain sensitivity once out from quiescence (128). In patients with chronic myeloid leukemia and non-small cell lung cancer, ablation of F-box/WD repeat-containing protein 7 (FBXW7), a ubiquitin ligase that regulates dormancy by degrading cMyc and Notch (291), pushes CSCs out of dormancy and thus significantly enhances the benefit of imatinib and gefitinib, respectively (292, 293). Likewise, human leukemia stem cells efficiently exit the quiescent state and enter an active cell cycle following the administration of granulocyte colony-stimulating factor (GCSF) and IFN-α (294, 295). Proliferating stem cells are then vulnerable to cytarabine- and 5-fluoro-uracil-based chemotherapy (294, 295).

In a more recent study, inhibition of macroautophagy could force quiescent ovarian CSCs out of G_0_ and prevent further entry into quiescence (296). The dependence on specific niches (see above) represents a therapeutic opportunity for preventing or reducing metastasis outgrowth. This is exemplified by the targeting of E-selectin- and SDF-1 in the bone perivascular metastatic niche, which disrupts the anchorage of dormant breast cancer cells (160). This forces the mobilization of dormant cells into the bloodstream, where they are more vulnerable to chemotherapy, thus preventing metastatic colonization. Along similar lines, breaking the foothold of dormancy by targeting blood vessels, the ECM, or effector immune cells may prove effective in inhibiting dormant cancer cell survival and eventually relapse (297–299). Indeed, the blockade of the CCL2-C-C Motif Chemokine Receptor 2 (CCR2) axis, involved in breast cancer cell metastatic seeding in the lungs and recruitment of metastasis-associated macrophages (300), has provided therapeutic benefit in fibrosarcoma models (301). Similarly, inhibition of neutrophil infiltration by targeting the Notch1 signaling prevented lung metastatic spread of breast, ovary, and colorectal carcinoma, as well as melanoma (302). Overall, these pieces of evidence may offer new opportunities to specifically target DCCs and strategically eliminate MRD.

Data from clinical trials are emerging, and the results are promising (Table 2). As an example, in breast cancer patients docetaxel treatment following adjuvant fluorouracil, epirubicin, and cyclophosphamide (FEC) therapy successfully erased dormant DCCs (as detected in BM aspirates) while increasing the rates of metastasis-free survival [(303) NCT00248703]. Moreover, multiple on-going trials are exploiting immunotherapeutic protocols to target dormant cells. To reach a successful outcome, a few parameters have to be properly addressed. First, as dormant cancer (stem) cells develop early during tumor progression, their antigenic cargo is relatively poor. This, coupled with a reduced capability to present antigen on MHC-I, renders dormant cells poorly immunogenic. Alternative strategies based on chimeric antigen receptor (CAR) T (304) and NK cells (305) can be developed to overcome these limitations. Second, the high intra- and inter-patient heterogeneity of most tumors represents an additional challenge, that could be only addressed with cost-prohibitive personalized protocols. However, preclinical studies have shown the possibility of rapidly and easily reprogram circulating T cells *in situ* (306). Third, not all patients presenting DCCs in BM aspirates *de facto* develop metastases (137), and so it is of utmost importance to identify the additional parameters that characterize high-risk patients, thus avoiding the over-treatment of low-risk patients.

***Lock-in*** strategies aim at artificially keeping cancer cells in a dormant state, thus preventing their outgrowth (138). To date, the Adjuvant Tamoxifen: Longer Against Shorter (ATLAS NCT00003016) is the most significant trial that has used a strategy specifically based on forcing dormancy maintenance (Table 2). In this clinical trial, ER positive breast cancer patients showed a significant reduction of disease recurrence and metastasis outgrowth, when the standard 5 year adjuvant tamoxifen administration was extended to 10 years (307, 308).

A plethora of signaling pathways previously identified as regulators of cancer cell quiescence in preclinical studies can be exploited as potential therapeutic targets. Specifically, two strategies can be conceived. The first strategy is based on the activation of dormancy-maintaining factors. Thus, the activation of the stress-activated protein kinase p38 was shown to preserve a state of protracted dormancy in different cancer types (99, 309). Similarly, in breast cancer models, induction of the morphoregulatory gene Homeobox (Hox)D10 reverted tumorigenic cells into a growth-arrested phenotype (310, 311). The same effects are ascribed to the multiple microenvironmental factors described above, which drive quiescence by triggering low-mitogenic and high-stress signaling. For instance, metastasis-incompetent primary tumors promoted the conversion of recruited myeloid cells from pro- to anti-metastatic by forcing them to produce the antitumorigenic factor thrombospondin-1 (312). Moreover, stromal BMP7 triggered dormancy of prostate CSCs by activating p38, inducing the cell cycle inhibitor p21, and the metastasis suppressor NDRG1 (100), which is in line with the evidence that the inhibition of BMP4 reawakened dormant breast CSCs and favored lung colonization (150). Finally, the TGF-β2 signaling was also involved in the maintenance and/or induction of a quiescent state for BM DCCs in a head and neck squamous cell carcinoma model (147).

A second strategy is based on the chronic silencing of reawakening pathways. Specifically, a blockade of uPAR affected the FN1-dependent mitogenic signaling, resulting in a lack of ERK1/2 activity and induction of dormancy in head and neck squamous cell carcinoma cells (173, 309). Similarly, suppression of MAPK/ERK axis and SFK signaling, favored quiescence in breast cancer models (176, 313). Also, the inhibition of the lysophosphatidic acid receptor 1 (LPAR1) induced dormancy of breast metastatic lesions by activating p38 signaling (74). The DNA methylation inhibitor 5-azacytidine interrupted the G_0_ → G_1_ switch in leukemia and breast cancer cells (314). In a subsequent study, the same authors showed that a combination of 5-azacytidine with bortezomib induces long-term dormancy multiple myeloma cells (315).

The advent of omics-based approaches disclosed single cell snapshots of molecular signatures associated with cancer dormancy (106, 316–318), some of which represent every promising target. Although theoretically highly attractive and clinically highly beneficial, the idea of keeping cancer (stem) cells asleep, may be difficult to translate into clinical settings. Some patients with a good prognosis and no more evidence of disease may be reluctant to continue therapy indefinitely. Moreover, long-term follow-ups and accumulating costs are additional challenges that need to be carefully considered. Interestingly, screening of the Prestwick Library, made up of Food and Drug Administration (FDA) approved drugs, led to the identification of the stimulant laxative drug bisacodyl as the sole agent specifically inhibiting quiescent, but not proliferating, glioblastoma stem-like cells (319). This opens the avenue to a third therapeutic strategy: the targeting of cancer (stem) cells while they are dormant. Intense basic and clinical research is developing, for example, target therapy with ABT-737, an inhibitor of anti-apoptotic BCL2 family members exerted a robust and preferential cytotoxic activity on quiescent lung CSCs (320). These findings opened the possibility to combine conventional chemotherapy with ABT-737 to kill otherwise resistant dormant CSCs, and thus prevent their relapse after reawakening (320).

We urge that more studies further explore dormancy regulation. These future studies will offer new possibilities for marker detection and metastatic prediction, opening a therapeutic window for prevention trials.

## Concluding Remarks

This is an exciting moment for cancer research, with data bringing into sharp focus the complex factors and mechanisms that render the TME either metastasis-permissive or metastasis-suppressive, but we still have a long way to go. The ability to anticipate whether, when, and how dormant DCCs are reactivated could help make cancer curative intent a reality. The striking analogies between dormant DCCs and dormant CSCs, along with their co-evolution with the surrounding microenvironment, may provide the ground for developing therapies that consider dormancy as a whole process. This opportunity to rethink therapeutic strategies could be the way to eradicate and/or prevent lethal metastatic recurrence and would surely benefit from the possibility of monitoring dormancy over time through rigorous, non-invasive, and preferably low-cost approaches.

## Author Contributions

AS and RDM conceived the paper. AS wrote the first version of the manuscript and designed the figures with the help of MM, under the supervision of RDM. AS, MM, and CG prepared the tables, under the supervision of RDM. IV provided critical input to the preparation of the paper. All authors approved the final version of the article.

## Conflict of Interest

RDM is an advisory board member and research grant recipient from Hibercell, Inc. NY. The remaining authors declare that the research was conducted in the absence of any commercial or financial relationships that could be construed as a potential conflict of interest.
